# Staphylococci and fecal bacteria as bioaerosol components in animal housing facilities in the Zoological Garden in Chorzów

**DOI:** 10.1007/s11356-021-14594-y

**Published:** 2021-06-01

**Authors:** Jacek Grzyb, Krzysztof Pawlak

**Affiliations:** 1 Department of Microbiology and Biomonitoring, University of Agriculture in Kraków, Mickiewicza Ave 24/28, 30-059 Kraków, Poland; 2 Department of Zoology and Animal Welfare, University of Agriculture in Kraków, Mickiewicza Ave 24/28, 30-059 Kraków, Poland

**Keywords:** Bioaerosol exposure, Occupational exposure, Staphylococci, Fecal bacteria, Antibiotic resistance

## Abstract

Zoos are places open for a large number of visitors, adults and children, who can admire exotic as well as indigenous animal species. The premises for animals may contain pathogenic microbes, including those exhibiting antibiotic resistance. It poses a threat to people remaining within the zoo premises, both for animal keepers who meet animals on a daily basis and visitors who infrequently have contact with animals. There are almost no studies concerning the presence on the concentration of airborne bacteria, especially staphylococci and fecal bacteria in animal shelters in the zoo. There is no data about antibiotic resistance of staphylococci in these places. The results will enable to determine the scale of the threat that indicator bacteria from the bioaerosol pose to human health within zoo premises. This study conducted in rooms for 5 animals group (giraffes, camels, elephants, kangaroos, and Colobinae (species of monkey)) in the Silesian Zoological Garden in Chorzów (Poland). The bioaerosol samples were collected using a six-stage Andersen cascade impactor to assess the concentrations and size distribution of airborne bacteria. Staphylococci were isolated from bioaerosol and tested for antibiotic resistance. In our study, the highest contamination of staphylococci and fecal bacteria was recorded in rooms for camels and elephants, and the lowest in rooms for Colobinae. At least 2/3 of bacteria in bioaerosol constituted respirable fraction that migrates into the lower respiratory tract of the people. In investigated animal rooms, the greatest bacteria contribution was recorded for bioaerosol fraction sized 1.1–3.3μm. Bacterial concentrations were particularly strong in spring and autumn, what is related to shedding fur by animals. Among the isolated staphylococci which most often occurred were *Staphylococcus succinus*, *S. sciuri*, and *S. vitulinus*. The highest antibiotic resistance was noted in the case of *Staphylococcus epidermidis*, while the lowest for *S. xylosus*. In addition to standard cleaning of animal rooms, periodic disinfection should be considered. Cleaning should be carried out wet, which should reduce dust, and thus the concentrations of bacteria in the air of animal enclosures.

## Introduction

Zoos are places where a large number of visitors, adults and children, can admire both exotic and native species of animals. Animals and their surroundings are sources of bioaerosol. It is expected that in the case of animals kept in closed facilities, bioaerosol concentrations will be higher compared to free range. The environment inside the premises intended for animals contains pathogenic microbes, including those exhibiting antibiotic resistance (Górny and Dutkiewicz 2002). It poses a threat to people remaining within the zoo premises, both for animal keepers who meet animals on a daily basis and visitors who infrequently have contact with animals. Bioaerosol exposure may cause more serious effects in young children compared to adults, what is connected with distinct structure of their respiratory tract, inhaling greater quantity of air in relation to their body weight, increased mobility, and not fully developed immune system (Choo and Jalaludin 2015).

Animals represent the largest source of microbiological contamination inside the facilities intended for them, specially their fur that is capable of transferring pathogens (Jo and Kang 2006). When it comes to that matter, particular attention should be directed to staphylococci that are found on skin, fur, epithelium, and mucous membranes, notably in moist areas (e.g., nose) (Irving et al. 2008).

The other sources of germs that may constitute bioaerosol include feed scraps, feces (remaining within the facilities for some part of the day), and litter. The last two sources may contain fecal bacteria. The amount of airborne fecal bacteria is also influenced by the size of the animal, the amount of feces produced by the animal, the consistency of the feces, and the height from which the feces fall to the ground. The fecal bacteria constituting bioaerosol can cause health problems (de Rooij et al. 2019).

Numerous studies demonstrated that contamination of the internal environment is correlated with the occurrence of both acute and chronic health problems (Samadi et al. 2013). The most frequently reported health issues regarded the respiratory system (rhinitis, bronchitis, sinusitis, asthma) and involved gastrointestinal disorders (Farthing et al. 2009; Borlée et al. 2015; Walser et al. 2015; Douglas et al. 2018; Robertson et al. 2019).

Taking the above into consideration, it is necessary to ensure safe conditions for people remaining in close proximity to animals with safe conditions in terms of microbial components. Unfortunately, there are no guidelines that would directly determine the microbiological quality of air for specific environments such as animal facilities in the zoo. Zoos are obliged to ensure proper welfare conditions (Kruszewicz 2011), as set forth in the Council Directive 1999/22/EC and the ordinance of the Minister of Environment of 2004 (J.L. of 2004, No. 5, item 32).

The purpose of the study conducted in selected animal facilities in the Silesian Zoological Garden in Chorzów was the following:

- To estimate the contamination with indicator bacteria—staphylococci and fecal bacteria
- To determine whether the concentration of total (TC) and respirable fraction (RF) of the bioaerosol differs depending on the group of studied animals
- To determine the distribution of aerodynamic diameters of the bioaerosol containing indicator bacteria
- To isolate staphylococci strains and determine its species
- To assess resistance to antibiotics of isolated staphylococci strains

The results will enable to determine the scale of the threat that indicator bacteria from the bioaerosol pose to human health within zoo premises.

This study is a continuation of previous experiments carried out in the zoo in Kraków: in the first, focused on bacterial bioaerosol (Grzyb and Lenart-Boroń 2019); in the second, focused on fungal bioaerosol (Grzyb and Lenart-Boroń 2020). The third work again concerns bacterial bioaerosol, but in a different facility: at the zoo in Chorzów (Grzyb and Pawlak 2021). In the present work, the indicator bacteria will be introduced as ingredients of bioaerosols. Staphylococci are a specific indicator in bioaerosols in animal environments, and fecal bacteria due to fecal contamination occurring in such places. No publications on this matter were found in the available literature, so the authors decided to explore this topic.

## Materials and methods

The study was conducted in the Silesian Zoological Garden in Chorzów (Poland). The zoo is located 272 m above the sea level, and its area amounts to 47.62ha. The object is situated on almost flat area.

The measurements were taken throughout the whole calendar year—twice each season. Within the season, the tests were carried out on days with similar microclimatic conditions.

The study involved rooms for the following animals: elephants (*Elephas maximus*), giraffes (*Giraffa camelopardalis reticulata*), kangaroos (*Macropus rufus*), camels (*Camelus bactrianus*), and monkeys called Colobinae (*Colobus guereza*). The animals remained within their premises during cold months, and could walk into enclosures during warm months. The research facilities were selected based on the following criteria: the size of animals (large (giraffes, elephants, camels) vs. small (Colobinae and kangaroos)) and the age of the animal facilities (new (for giraffes and Colobinae) vs. old (for elephants, kangaroos, and camels)).

**Fig. 1:**
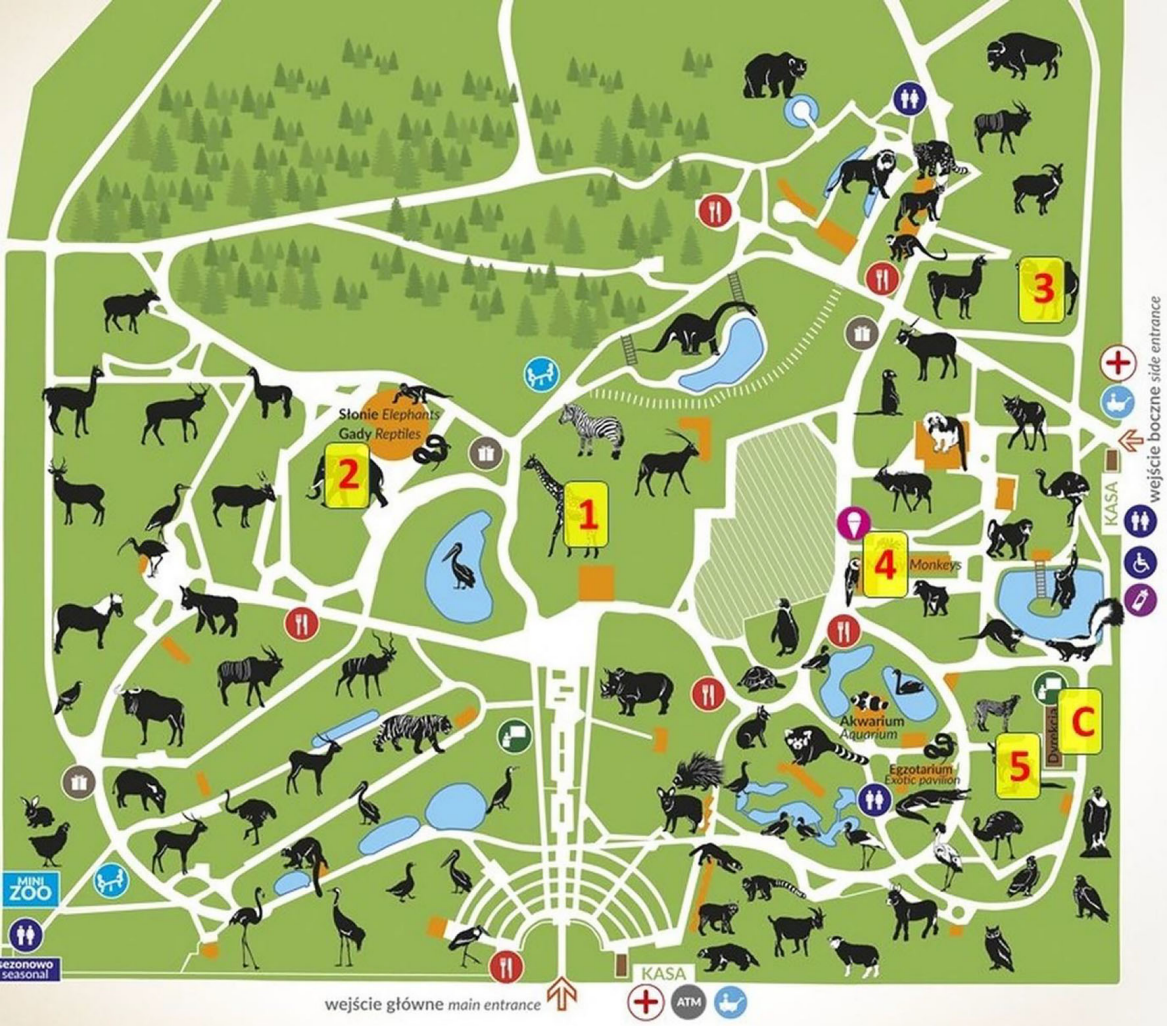
Location of the sampling sites nowy schemat. Legend: Shelters for **1** giraffes, **2** elephants, **3** camels, **4** Colobinae (monkeys), and **5** Kangaroos; C control

Location of the sampling sites is shown in Fig. 1 and their characteristics are shown in Table 1.

**Table 1:** Characteristics of the studied premises

Parameter	Group of animals				
	Elephants (*Elephas maximus*)	Camels (*Camelus bactrianus*)	Colobinae (*Colobus guereza*)	Giraffes (*Giraffa camelopardalis reticulata*)	Kangaroos (*Macropus rufus*)
Total area [m^2^]	200	50	38	343	25
Year of construction	1960s	1960s	2010	2013	1960s
Number of animals	2	2	10	5	5
Area per 1 animal [m^2^]	100	25	3.8	68.6	5
Type of ventilation	Lack	Lack	Mechanical	Mechanical	Lack
Mean animal weight [kg]	3500	480	14	1,500	47
Ratio—kg of animal weight per 1 m^2^ of area	35	19.2	3.7	21.9	9.4
Type of litter	Lack	Sawdust beech	Lack	Sawdust beech	Sawdust beech

The sampling sites with animals were inside buildings (I), in the middle of the animal space. The sampling site outside (O) was located on the parking, 5 m from the front of the office building.

Air samples were taken using a 6-stage cascade impactor WES-710 model Andersen-Graseby (Westech Instrument, Great Britain). This impactor enables to determine bioaerosol fractions based on the aerodynamic particle size: fraction 1, above 7μm; fraction 2, 4.7–7μm; fraction 3, 3.3–4.7μm; fraction 4, 2.1–3.3μm; fraction 5, 1.1–2.1μm, and fraction 6, 0.65–1.1μm. Fractions 3–6 (below 4.7μm) are classified as respirable**.** It is worth knowing that with the impactor sampler, only the number of particles carrying microorganisms can be determined and not all microorganisms in the respective particle size fraction. Regardless of whether there are several dozen bacterial cells on an impacted particle or only one, usually a one macroscopically visible colony grows on the microbial medium.

The samples were taken about the same time (between 10.00am and 1.00pm). At this time, animals have already been fed and their boxes were cleaned. Air samples were collected 1.5m above the ground, what is tantamount to the location of the human breathing zone. Six Petri dishes were used to collect the samples—one for each impactor stage. The time necessary to collect the samples depended on anticipated concentrations of bacteria in a given location. The flow rate through the impactor was constant and amounted to 28.3l/min. The samples were collected within 20 to 180 s, and the volume of aspirated air ranged between 9.4 and 84.9l. The impactor was disinfected using gauze pads moisten in 70% isopropanol before taking each sample.

The following microbial media were used in the study: Mannitol Salt Lab-Agar called Chapman medium (Biomaxima) for isolating staphylococci and EMB (Eosin Methylene Blue Agar; Biomaxima) for growing fecal bacteria—Enterobacteriaceae (*Escherichia coli*, *Klebsiella*, *Enterobacter*, *Salmonella*, and *Shigella*).

After sampling, the Petri plates were immediately transported to the laboratory, where they were incubated at 36±1°C for 48h. After the incubation, the colonies were counted and the results were expressed as colony forming units per 1m^3^ of air (CFU/m^3^). The concentrations of bacteria was calculated according to the formula: L = [Pr 1000]/v, where L is the concentration of microorganisms in 1 m^3^ of air, Pr is the probable statistical count according to the impactor manufacturer's table (Pr is read from the table on the basis of the number of colonies), v - volume of air taken by the impactor (dm^3^), and 1000 is the converter to 1 m^3^. The tests were performed in triplicate and the results were presented as the means.

As the guidelines on the acceptable concentrations of bioaerosol inside facilities intended for animals have not been developed yet, the results obtained in this study were evaluated against the values proposed by the Team of Experts in Biological Factors (Polish: ZECB) (Augustyńska and Pośniak 2016), with animals’ rooms being classified as working premises contaminated with organic dust (Table 2).

**Table 2:** Proposals for acceptable concentrations of airborne microorganisms in the working environment according to the Team of Experts in Biological Factors (ZECB) – the values applicable in Poland

Microbiological agent	Acceptable concentration [CFU/m^3^]
Gram-negative bacteria (total count, TC)	20,000
Gram-negative bacteria (respirable fraction, RF)	10,000
Staphylococci	No data

Staphylococcal strains were identified according to the methodology devised by Lenart-Boroń et al. (2016). Pure staphylococcal strains were obtained from the colonies growing on Chapman medium by means of streak plating. Pure strains were subjected to microscopic examination and furazolidone susceptibility testing. Micrococci were not included in the study due to their resistance to furazolidone, while sensitive strains were classified as staphylococci. Staphylococcal species were identified using a MALDI-TOF method. MALDI-TOF is a method for the identification of microorganisms based on the technique of mass spectrometry using laser desorption/ionization, supported by a matrix with a time-of-flight analyzer. A MicroFlex LT mass spectrometer from Bruker was used. The system runs under the FlexControl program. As a result of the analysis of the sample of the tested bacteria in the above-mentioned device, a mass spectrum is created with a characteristic pattern of mass and intensity distribution for a given microorganism, which is compared with the library of spectra of known microorganisms. Then the logarithm of the obtained result is calculated; if the identification index is ≥2, the identification is considered obtained with high certainty. The database of used patterns attached to the equipment is approximately 4500 species (Zieliński and Rajca 2018; Azarko and Wendt 2011).

The sensitivity of staphylococci to antibiotics was evaluated using paper disks immersed in selected antibiotic solutions. The antibiotics listed hereafter were used in the study: cefoxitin (FOX 30 μg), chloramphenicol (C 30 μg), ciprofloxacin (CIP 5 μg), fusidic acid (FA 10 μg), gentamycin (CN 10 μg), tigecycline (TGC 15 μg), erythromycin (E 15 μg), clindamycin (DA 2 μg), tetracycline (TE 30 μg), and rifampicin (RA 5 μg). Paper disks were transferred to Mueller-Hinton medium (Biomaxima Poland). Antibiotic resistance was eventuated according to the guidelines provided by the National Reference Center for Antimicrobial Susceptibility (Polish: KORLD) (Żabicka and Hryniewicz 2010) and EUCAST (Hryniewicz 2014).

Statistical analysis was performed using the Statistica 13.1 (StatSoft, USA). The values obtained for the concentrations of bacterial bioaerosol were expressed as means with standard deviations and range. Normal distribution of data was examined applying the Shapiro-Wilk test.

The distribution of total (TC) and respirable (RF) fraction of bioaerosol values was close to normal and other data were not normally distributed; therefore, both parametric (a one-way ANOVA followed by the post-hoc Tukey’s test – for α=0.05; Bulski et al. 2019) and non-parametric (the Kruskal-Wallis test; Frączek and Górny 2011) tests were applied to assess the significance of differences between the concentrations of bioaerosols in rooms for different animals.

## Results

The normative values were determined only for fecal bacteria. The concentrations calculated for this group (Table 3) were within the range of 0 to 1060 CFU/m^3^ and do not exceed values recommended by ZECB (see Table 2). As compared with acceptable concentration, maximum concentrations for fecal bacteria amounted to approx. 5% for TC and 10% for RF.

**Table 3:** Average, standard deviation, and range of bioaerosol of indicator bacteria in animal premises in Chorzów zoological garden [CFU/m^3^]

Group of animals	Fractions	Mannitol-positive staphylococci (ST-POS)	Mannitol-negative staphylococci (ST-NEG)	Fecal bacteria (FB)
Giraffes	TC	3499d ± 3100 (636–7635)	3128d ± 1040 (1908–4028)	70bc ± 99 (0–212)
	RF	2933d ± 2680 (636–6504)	2438c ± 966 (1767–3816)	70b ± 99 (0–212)
Elephants	TC	1899c ± 1373 (283–3286)	18,402e ± 34,244 (472–69,748)	435d ± 506 (0–1060)
	RF	1431c ± 1231 (94–2968)	16,132e ± 30,594 (212–62,010)	418c ± 498 (0–1060)
Camels	TC	6251e ± 3135 (3304–9827)	6539d ± 6452 (1416–15,978)	52b ± 105 (0–211)
	RF	4041e ± 1543 (2312–5726)	5332d ± 6057 (896–14,210)	52b ± 105 (0–211)
Colobinae	TC	1009b ± 1083 (105–2332)	736b ± 1023 (70–2261)	47b ± 66 (0–141)
	RF	630b ± 811 (0–1766)	400a ± 643 (70–1366)	11a ± 23 (0–47)
Kangaroos	TC	5902e ± 6630 (0–12,225)	1837c ± 2597 (282–5724)	123c ± 105 (0–212)
	RF	3834e ± 4458 (0–8978)	1254b ± 1757 (282–3886)	88b ± 88 (0–212)
Control	TC	133a ± 114 (0–275)	93a ± 59 (23–162)	0a ± 1 (0–2)
	RF	93a ± 112 (0–254)	78a ± 56 (11–148)	0a ± 0 (0–0)

Legend: The different letters within a column indicate a significant difference at p<0.05 according to Tukey’s test

Table 3 shows mean bioaerosol concentrations with standard deviations and concentration ranges. The highest bioaerosol concentration was obtained for mannitol-negative staphylococci (ST-NEG), lower for mannitol-positive staphylococci (ST-POS), and the lowest for fecal bacteria (FB). The mean concentrations for ST-POS and ST-NEG differ by up to one order of magnitude, while for ST-NEG and FB by, two orders of magnitude. The highest mean concentration for ST-POS, both for TC and RF, was detected in shelters for camels—it amounted to 6251±3135CFU/m^3^ and 4041±1543CFU/m^3^, respectively. The largest mean concentration for ST-NEG (TC: 18,402±34,244CFU/m^3^, RF: 16,132±30,594CFU/m^3^) and FB (TC: 435±506CFU/m^3^, RF: 418±498CFU/m^3^) was recorded in rooms for elephants. The lowest mean bioaerosol concentrations for 3 studied bacterial groups were measured in premises for Colobinae (ST-POS TC: 1009±1083CFU/m^3^, RF: 630±811CFU/m^3^; ST-NEG TC: 736±1023CFU/m^3^, RF: 400±643CFU/m^3^; FB TC: 47±66CFU/m^3^, RF: 11±23CFU/m^3^).

Regarding bioaerosol concentration broken down by fractions established based on aerodynamic particle size (Table 4), the highest bioaerosol concentration for ST-POS was detected in rooms for camels for the fraction 2.1–1.1μm (1909CFU/m^3^), for ST-NEG for the same fraction but in the facilities for elephants (5765CFU/m^3^). The highest concentration for FB was also detected in elephant facilities but concerned larger particle size—3.3–2.1μm (5686CFU/m^3^). After summing up all generated results for all studied bacterial groups, the share of individual bioaerosol factions can be arranged in the descending order: 2.1–1.1μm > 3.3–2.1μm >4.7–3.3μm>1.1–0.65μm>11–7μm>4.7–7μm. The highest bacteria concentrations were detected in fractions classified as respirable, as confirmed by the data shown in Table 5. Depending on the bacterial group, season, and facilities intended for a given animal species, the share of respirable fraction ranged from 0 to 100%. One hundred percent RF was achieved most frequently for fecal bacteria—e.g., in spring in rooms for giraffes, elephants, Colobinae, and kangaroos. That relationship results from the fact that fecal bacteria form large consortia, consisting of bacteria alone or bacteria attached to dust particles, less frequently than staphylococci. The average share of FR in TC, taking into account all studied animal facilities and 3 bacterial groups, was the largest in winter (74.28%), while the lowest in summer (65.93%). The analysis of the results for particular season indicated that the above-mentioned pattern can be directly connected with time spent by animals inside their rooms that is undoubtedly the longest in winter. As far as the animal groups are concerned, the greatest RF share amounting to 85.35% was recorded in shelters for giraffes, while the lowest in rooms for kangaroos. There was no distinct relationship between the RF share and any of the factors.

**Table 4:** Fraction average concentrations of bioaerosol of indicator bacteria in animal premises in Chorzów zoological garden [CFU/m^3^]

Fraction of bioaerosol [μm]	Group of bacteria	Group of animals					
		Giraffes	Elephants	Camels	Colobinae	Kangaroos	Control
11–7	ST-POS	247a	183a	1161b	280a	866b	19a
	ST-NEG	424ab	1063b	648b	91a	424ab	8a
	FB	0a	0a	0a	24a	35a	0a
7–4.7	ST-POS	318ab	286ab	1049b	100a	1202c	21a
	ST-NEG	265ab	1207c	560b	244ab	159a	8a
	FB	0a	18a	0a	12a	0a	1a
4.7–3.3	ST-POS	636b	256ab	1102b	135a	1060b	18a
	ST-NEG	424b	2541c	790b	138a	495b	18a
	FB	0a	88a	0a	0a	0a	0a
3.3–2.1	ST-POS	636b	439b	666b	253a	1396c	29a
	ST-NEG	548b	5686c	943bc	132a	389b	31a
	FB	0a	247b	35a	0a	0a	0a
2.1–1.1	ST-POS	866ab	286a	1909b	171a	1149b	24a
	ST-NEG	760b	5765d	1868c	47a	177a	19a
	FB	53b	82b	0a	0a	0a	0a
1.1–0.65	ST-POS	795b	451b	365ab	71a	230ab	21a
	ST-NEG	707b	2141c	1732c	82a	194a	10a
	FB	18a	0a	18a	12a	88a	0a

Legend: The different letters within a row indicate a significant difference at p<0.05 according to Tukey’s test

**Table 5:** Percentage share of respirable fraction (RF) depends on season [%]

Season	Group of bacteria	Group of animals						Average share for season
		Giraffes	Elephants	Camels	Colobinae	Kangaroos	Control	
Spring	ST-POS	66.7ab	90.3b	58.3ab	66.7ab	60.0ab	23.1a	71.11a
	ST-NEG	60.7ab	88.9b	88.9b	28.6a	55.6ab	50.0ab	
	FB	100.0a	100.0a	NA	100.0a	100.0a	NA	
Summer	ST-POS	86.0a	64.1a	61.6a	75.8a	50.3a	77.8a	65.93a
	ST-NEG	94.7b	30.0a	54.4a	100.0b	67.9a	70.0a	
	FB	NA	88.9b	100.0b	NA	33.3a	0.0a	
Autumn	ST-POS	85.2b	33.3a	70.0b	0.0a	81.4b	NA	69.70a
	ST-NEG	67.6b	80.0b	63.3b	25.0a	70.0b	100.0b	
	FB	100.0a	100.0a	NA	NA	100.0a	NA	
Winter	ST-POS	100.0b	70.4b	82.1b	45.2a	NA	92.3b	74.28a
	ST-NEG	92.6ab	71.9ab	85.1ab	60.4a	100.0b	91.3ab	
	FB	NA	NA	NA	0.0a	NA	NA	
Average share RF for group of animals		85.35b	74.35b	73.74b	50.17a	71.85b	63.06a	

Legend: The different letters within a row indicate a significant difference at p<0.05 according to Tukey’s test

*ST-POS*, mannitol-positive staphylococci; *ST-NEG* mannitol-negative staphylococci; *FB* fecal bacteria; *NA* not applicable

**Fig. 2:**
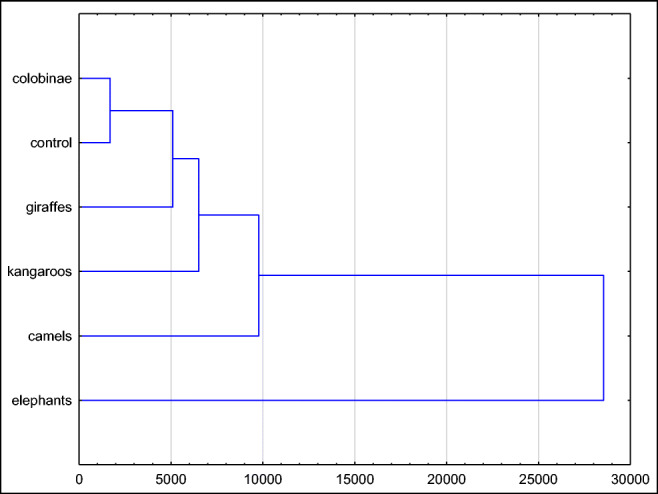
Cluster analysis for shares of fractions of bacterial bioaerosol in animal shelters in the zoo in Chorzów

The results of cluster analysis for bioaerosol concentrations in rooms for different animals are shown in Fig. 2. As presented in Fig. 2, the parameters obtained for Colobinae facilities show the greatest similarity to those recorded in the control area. It means that parameters in rooms for Colobinae are comparable to the ones recorded in the external environment. The environment in the facilities for elephants differed considerably from the control site and remaining animal rooms.

The indoor/outdoor ratio (I/O ratio) that is the ratio between bioaerosol concentration indoors and outside is an important indicator of microbial air pollution. When I/O ratio is higher than 1, it means that the room has been intoxicated. Table 6 shows I/O ratios arranged from the lowest to the highest values, separately for 3 studied bacterial groups, including TC and RF. The greatest intoxication occurred in spring and autumn, especially in the case of ST-POS, what can be easily explained as staphylococci constitute natural skin, fur, and mucous membrane microflora. The lowest indoor intoxication with staphylococci and fecal bacteria was recorded in winter, what might be related to low animal activity. In the case of animals shedding fur, winter is a stable period. However, the situation changes in spring and autumn, when fur is shed: patches of shed fur facilitate bacterial growth, including staphylococci (predominantly) and fecal bacteria (when the animal lies in feces that has not been cleaned).

**Table 6:** Dependence ratio I/O on group of animals/season for total concentration of bacterial bioaerosol (TC) and respirable fraction of bacterial bioaerosol (RF)

Group of bacteria					
ST-pos		ST-neg		FB	
I/O for TC	I/O for RF	I/O for TC	I/O for RF	I/O for TC	I/O for RF
0a K/win	0a Cl/aut	1a Cl/sum	1a Cl/sum	0a G/sum	0a G/sum
1a Cl/spr	0a K/win	2a K/win	1a Cl/aut	0a G/win	0a G/win
2a G/win	3a G/win	4a Cl/aut	2a K/win	0a E/win	0a E/win
2a K/spr	3a Cl/spr	6a E/sum	3a E/sum	0a Cm/spr	0a Cm/spr
5a Cl/win	3a Cl/win	7a E/aut	5a E/aut	0a Cm/aut	0a Cm/aut
5a E/win	4a E/win	10a K/aut	7a K/aut	0a Cm/win	0a Cm/win
11a G/spr	6a K/spr	12a G/win	8a Cl/spr	0a Cl/sum	0a Cl/sum
14a Cm/win	13a Cm/win	14a Cl/spr	9a Cl/win	0a Cl/aut	0a Cl/aut
21a E/spr	21a E/sum	14a Cl/win	12a G/win	0a K/win	0a Cl/win
22a Cl/sum	21a Cl/sum	17a E/win	13a E/win	47a E/aut	0a K/win
26a E/sum	32a G/spr	20a Cm/aut	13a Cm/aut	47a Cl/spr	47a E/aut
38a G/sum	42a G/sum	27a K/spr	25a G/aut	71a G/aut	47a Cl/spr
64a Cm/spr	59a Cm/sum	29a Cm/win	27a Cm/sum	71a K/spr	71a G/aut
75a Cm/sum	75a K/sum	34a G/sum	27a Cm/win	106a Cm/sum	71a K/spr
106a Cl/aut	84a E/spr	34a Cm/sum	30a K/spr	106a K/sum	71a K/sum
115b K/sum	94a E/aut	37a G/aut	46a G/sum	141ab Cl/win	212ab G/spr
283b E/aut	162a Cm/spr	49a K/sum	47a K/sum	212b G/spr	212ab Cm/sum
3304c Cm/aut	2313b Cm/aut	168a G/spr	204ab G/spr	212b K/aut	212ab K/aut
7636c G/aut	6504ab G/aut	677b Cm/spr	1204b Cm/spr	318b E/sum	565b E/sum
11,029c K/aut	8979b K/aut	2955b E/spr	5255b E/spr	1060b E/spr	1060b E/spr

Legend: The different letters within a column indicate a significant difference at p<0.05 according to Tukey’s test—between groups of animals; *G* giraffes, *E* elephants, *Cm* camels, *Cl* Colobinae, *K* kangaroos, *spr* spring, *sum* summer, *aut* autumn, *win* winter

This study also involved identification of staphylococcal species occurring in rooms intended for animals in the zoo (Table 7). *Staphylococcus succinus* was the most frequently found bacteria. It constituted almost 1/3 of all isolated staphylococcal strains. *S. sciuri* was the second most frequently occurring bacteria (19.1%) and *S. vitulinus* the third (12.6%). *S. haemolyticus* was the least frequently detected among isolated staphylococcal species.

**Table 7:** The frequency of occurrence of staphylococci species in animal shelters in the zoo in Chorzów

Group of animals	Species of bacteria	Frequency of occurrence [%]
Giraffes	*Staphylococcus succinus*	34.8
	*Staphylococcus sciuri*	23.2
	*Staphylococcus xylosus*	17.4
	*Staphylococcus vitulinus*	16.2
	*Staphylococcus equorum*	7
	*Staphylococcus chromogenes*	1.4
Elephants	*Staphylococcus succinus*	34
	*Staphylococcus xylosus*	18.7
	*Staphylococcus sciuri*	17
	*Staphylococcus vitulinus*	15.3
	*Staphylococcus equorum*	9.9
	*Staphylococcus chromogenes*	1.7
	*Staphylococcus cohnii*	1.7
	*Staphylococcus epidermidis*	1.7
Camels	*Staphylococcus succinus*	29.9
	*Staphylococcus xylosus*	20.4
	*Staphylococcus sciuri*	17.7
	*Staphylococcus vitulinus*	12.2
	*Staphylococcus equorum*	5.4
	*Staphylococcus gallinarum*	4.2
	*Staphylococcus capitis*	4.1
	*Staphylococcus lentus*	3.3
	*Staphylococcus chromogenes*	2.7
Colobinae	*Staphylococcus succinus*	57
	*Staphylococcus vitulinus*	17.1
	*Staphylococcus epidermidis*	14.3
	*Staphylococcus gallinarum*	5.7
	*Staphylococcus capitis*	3.1
	*Staphylococcus haemolyticus*	2.9
Kangaroos	*Staphylococcus succinus*	40.3
	*Staphylococcus vitulinus*	15
	*Staphylococcus xylosus*	12.7
	*Staphylococcus sciuri*	11.5
	*Staphylococcus chromogenes*	6.8
	*Staphylococcus capitis*	4.6
	*Staphylococcus gallinarum*	4.6
	*Staphylococcus equorum*	3.5
	*Staphylococcus epidermidis*	1.2
Control	*Staphylococcus sciuri*	45.4
	*Staphylococcus cohnii*	27.2
	*Staphylococcus epidermidis*	13.6
	*Staphylococcus lentus*	9.1
	*Staphylococcus capitis*	4.7

The frequency of occurrence of individual staphylococcal species for different animal rooms is presented in Table 8. *S. succinus* occurred most frequently in all animal facilities and its share ranged from 29.9% in rooms for camels to 57% in facilities for Colobinae. The smallest share in the case of individual animal facilities was recorded for *S. chromogenes* (giraffes, camels), *S. epidermidis* (elephants, kangaroos), and *S. haemolyticus* (Colobinae). As regards the number of identified staphylococci species in animal rooms, it ranged from 6 (giraffes and Colobinae) to 9 (camels, kangaroos). Five staphylococci species were detected within the control area (outside animal premises).

**Table 8:** The total percentage of staphylococci species in animal shelters in the zoo in Chorzów

Species of bacteria	Frequency of occurrence [%]
*Staphylococcus succinus*	32.7
*Staphylococcus sciuri*	19.1
*Staphylococcus vitulinus*	12.6
*Staphylococcus xylosus*	11.5
*Staphylococcus epidermidis*	5.1
*Staphylococcus cohnii*	4.8
*Staphylococcus equorum*	4.3
*Staphylococcus capitis*	2.7
*Staphylococcus gallinarum*	2.4
*Staphylococcus chromogenes*	2.1
*Staphylococcus lentus*	2.1
*Staphylococcus haemolyticus*	0.5

Isolated staphylococci—100 strains belonging to 12 species—were subjected to antibiotic resistance testing. Ten antibiotics were used in the study. Antibiotic names and doses used to immerse paper disks are shown in Table 9. It has been established that studied bacterial strains showed the highest resistance to fusidic acid and rifampicin, and overall susceptibility to 5 antibiotics: ciprofloxacin, chloramphenicol, gentamycin, tigecycline, and erythromycin. The highest average resistance to tested antibiotics (Table 10) was recorded for strains belonging to *S. epidermidis* (22.5%), similar (approx. 20%) for *S. sciuri* and *S. lentus*, while the lowest for *S. xylosus* (4%).

**Table 9:** The total resistance of staphylococci strains isolated from the air in animal shelters in the zoo in Chorzów

Antibiotic name	Dose [mg]	Code	Share of strains resistant [%]
Fusidic acid	10	FA10	42
Rifampicin	5	RA5	36
Tetracycline	30	TE30	27
Clindamycine	2	DA2	22
Cefoxitin	30	FOX30	1
Ciprofloxacin	5	CIP5	1
Chloramphenicol	30	C30	0
Gentamycin	10	CN10	0
Tigecycline	15	TGC15	0
Erythromycin	15	E15	0

**Table 10:** Antibiotics resistance of staphylococci isolated from the air in animal shelters in the zoo in Chorzów

Species of staphylococci	Number of strains	Antibiotic code (see table 9)						Average resistance of the species [%]
		DA2	FA10	FOX30	RA5	TE30	C30, CIP5, CN10, E15, TGC15	
*Staphylococcus capitis*	1	0	0	0	0	100	0	10
*Staphylococcus cohnii*	3	0	33	0	0	33	0	6.6
*Staphylococcus chromogenes*	2	0	50	0	50	50	0	15
*Staphylococcus epidermidis*	4	25	100	25	50	25	0	22.5
*Staphylococcus equorum*	6	0	0	0	0	17	0	1.7
*Staphylococcus gallinarum*	6	50	100	0	0	0	0	15
*Staphylococcus haemolyticus*	1	0	100	0	0	0	0	10
*Staphylococcus lentus*	3	0	100	0	33	67	0	20
*Staphylococcus sciuri*	16	28	62	0	56	61	0	20.7
*Staphylococcus succinus*	30	29	29	0	42	32	0	13.2
*Staphylococcus vitulinus*	14	0	0	0	58	14	0	7.2
*Staphylococcus xylosus*	14	0	40	0	0	0	0	4

## Discussion

Available literature fails to provide reports on the presence and concentrations of staphylococci and fecal bacteria in very specific environments, such as animal facilities in the zoos. Another difficulty is concerned with the lack of normative values for staphylococci. As regards fecal bacteria, existing normative values were not exceeded. Recorded concentrations, as compared with normative values, amounted to approx. 5% for TC and 10% for RF.

The staphylococci concentrations recorded in this study conducted in the zoo in Chorzów ranged from 0 to 6.9×10^4^CFU/m^3^. The lowest concentrations were noted in winter, while the highest in spring. Also in winter, the lowest concentrations of staphylococci were recorded by Wolny-Koładka (2018a) while conducting research at horse riding centers (1.5×10^1^–2.56×10^3^CFU/m^3^). The highest concentrations of staphylococci were recorded by Wolny-Koładka in summer (2×10^2^–4.12×10^3^CFU/m^3^). As compared with the concentrations of staphylococci obtained by Wolny-Koładka, our results were slightly higher. Staphylococci count obtained by Masclaux et al. (2013) carrying out research on a pig farm in Switzerland fell within the following range: 1.9×10^3^–4×10^8^CFU/m^3^. However, in summer, the concentrations ranged from 1.9×10^3^ to 4.7×10^7^CFU/m^3^, while in winter, it increased by one order of magnitude (5.9×10^4^–4×10^8^CFU/m^3^). In our study, the concentrations for staphylococci were lower by at least three orders of magnitude, as compared with the results obtained by Masclaux et al.

In our study, the daily mean concentration for fecal bacteria amounted to 2.4×10^1^CFU/m^3^. Equally low concentrations were found at horse riding centers by Wolny-Koładka (2018a). It indicates that zoo keepers and horse keepers maintain proper cleanness standards in rooms intended for animals, what is surely associated with much lower stock as compared with large-scale farms.

The application of an Andersen-Graseby cascade impactor in the experiments enabled us to estimate the potential level of bioaerosol penetration into the human respiratory system based on the bacteria aerodynamic size (Górny et al. 2016). Madsen et al. (2018) used an Andersen cascade impactor to determine which bioaerosol fraction contains staphylococci based on the aerodynamic size of particles/aggregates formed by these bacteria. According to Madsen, 70% of bioaerosol contain staphylococci aggregates sized 7–11μm. It indicates that they are deposited in the upper respiratory tract, 22% of bioaerosol in the primary and secondary bronchi and 8% in the terminal bronchi and alveoli. Our study delivered contradictory results, fraction sized 7–11μm constituted only 11.2%, and the largest number of staphylococci was detected in fraction sized 2.1–1.1μm (27%). It means that this part of bioaerosol reaches terminal bronchioles. A significant part of bioaerosol (77.6%) was qualified as FR.

Clauß (2015) in a review article addressed the topic of the distribution of bioaerosol fractions for fecal bacteria and staphylococci. The data for fecal bacteria presented in that article are consistent with the results generated in this study. However, the results obtained for staphylococci were varying. In the above-cited article, the fraction above 4.7μm constituted more than 50%, while in our study as little as 22.4%.

As seen in various research, bioaerosol particles with the diameter lower than 2.5μm pose the most serious threat to the exposed people. This fraction is capable of penetrating into the lower pulmonary tract (to pulmonary alveoli), what often leads to health problems, such as low birth weight, heart and lung diseases, cancer, and premature death (Morakinyo et al. 2016). As claimed by Clauß (2015) in the case of rooms intended for animals, it may depend on many factors like room area, number of animals, animal size and weight, and the presence or absence of litter.

The research conducted previously in the same object (Grzyb and Pawlak 2021) revealed that I/O ratio for bacteria reached maximum value of 344 for TC and 785 for RF. In the case of indicator bacteria, ST-POS in particular, I/O ratio reached the value of 11,029 for TC and 8979 for RF. It means that the difference between staphylococci concentration indoors and in the outside air was high. Staphylococci contamination inside facilities for zoo animals can be easily explained, as staphylococci constitute natural fur, skin, and mucous membrane microflora (Friese et al. 2013; Feld et al. 2018; Kizerwetter-Świda and Pławińska-Czarnak 2017).

As results from the research carried out by Schulz et al. (2004), staphylococci can be used as a reliable and useful indicator for determining safe distance between rooms intended for animals and residential buildings as well as the spread of bioaerosol in the air surrounding animal shelters. They also found that the isolated staphylococcal species in the indoor and outdoor air were found on poultry skin and also in the litter. This indicates that skin particles and particles from the litter were the main source of airborne staphylococci. In turn, Duan et al. (2009) showed that 100% of strains of *Escherichia coli* from air samples collected in the pig house were similar to those isolated from pig feces.

The most burning issue relating to the antibiotic resistance is concerned with viewing staphylococci as a serious threat to humans and animals. The most frequent reports regarding the risks associated with staphylococci concern *Staphylococcus aureus* that can show resistance to methicillin (methicillin-resistant SA, MRSA) or vankomycin (vankomycin-resistant SA, VRSA). The information that majority of *S. aureus* strains are saprophytic and occur on human skin, animal fur, and mucous membranes is less popular. *S. aureus* can be found in 20–40% of human population. It colonizes nostrils and does not cause any health problems. However, *S. aureus* can cause opportunistic infections of the skin and soft tissues as well as inflammation of the entire body (sepsis). The incidence rate for staphylococci in the air samples depends on the sampling spot. Messi et al. (2015) carried out experiments in public places in Italy and reported that staphylococci constituted 17% of all isolated bacteria, while *S. aureus* strains approx. 1.7%. Our study covered 200 staphylococcal strains but *S. aureus* was not detected.

The studies undertaken by Ferguson et al. (2016) confirmed that disinfecting rooms for animals results in killing staphylococci, including MRSA. Thus when the concentration of staphylococci is high, it is advised to schedule periodic disinfection of animal rooms as an efficient measure reducing the number of undesired germs. It must be remembered that indicator bacteria analyzed in this study are potentially pathogenic. They migrate by means of direct transmission through dirty hands or orally, and as bioaerosol, what poses threat to animal keepers and—much lesser degree—to zoo visitors (Bos et al. 2016).

The presence of staphylococci indicates the possible inherence of pathogenic microorganisms, in which antibiotic resistance has been observed with increasing frequency over the past several decades (Małecka-Adamowicz et al. 2020). In this study *Staphylococcus succinus* was the most numerous staphylococcal species (approx. 33%), followed by *S. sciuri* (19.1%) and *S. vitulinus* (12.6%). Schulz et al. (2004) detected *S. saprophyticus*, *S. cohnii*, *S. arlettae*, and *S. lentus* on a broiler farm. Similar results were obtained by Popescu et al. (2011), who conducted research in stables. They identified two staphylococci species with more than 20% share in the entire number of isolated strains: *S. sciuri* and *S. xylosus*. Comparable species composition with the highest *S. sciuri* and *S. lentus* share was shown in Italian stables by De Martino et al. (2010). Contradictory results were delivered by Popescu et al. (2011), who established that *S. epidermidis* is the most numerous bacteria species with the share amounting to approx. 25%. In our study, it constituted 5% of all identified bacterial species. Haas et al. (2021) investigating pig barns also received conflicting results. The greatest share among all isolated staphylococcal species was recorded for *S. pasteuri* (47.9%) and *S. cohnii* subsp. *cohnii* (24.5%), while the lowest for *S. chromogenes* (1.06%).

The importance of antibiotic resistance on a global scale was considered very important by the World Health Organization (WHO). WHO states that without quick action, we will move to the post-antibiotic era (Małecka-Adamowicz et al. 2020). Antibiotic resistance is an important feature of staphylococci. It is the result of several mechanisms in staphylococcal cells: enzymatic inactivation of antibiotic molecules, active efflux, i.e. removal of antibiotics from the cell, change of drug affinity to the target site in the bacterial cell, reduction of the permeability of the bacterial cell membrane, and formation of bacterial mutations in progressive selection of resistant bacteria by antibiotics (Acar and Moulin 2012; Lenart-Boroń et al. 2017). The resistance to selected antibiotics found in our studies is consistent with the results obtained by Wolny-Koładka (2018b).

The antibiotics used in the experiment have a bacteriostatic effect on the tested staphylococcal strains, mainly by inhibiting the synthesis of proteins (fusidic acid, tetracycline, clindamycine, chloramphenicol, gentamycin, tigecycline, erythromycin), DNA synthesis (ciprofloxacin), RNA synthesis (rifampicin), and the synthesis of murein (cefoxitin) (Truszczyński et al. 2013). With reference to the data in Table 10, the tested antibiotics in most cases inhibited the synthesis of proteins in staphylococcal cells (fusidic acid, tetracycline, clindamycine) and RNA synthesis (rifampicin).

## Conclusions

In this study, the highest concentration of mannitol-positive staphylococci (ST-POS) was recorded in rooms for camels, while the greatest concentration of mannitol-negative staphylococci (ST-NEG) in shelters for elephants. The lowest concentrations for 3 analyzed bacterial groups were detected in rooms for Colobinae. Acceptable microbial concentrations for fecal bacteria were not exceeded.

In investigated animal premises, the greatest bacteria contribution was recorded for bioaerosol fraction sized 1.1–3.3μm. At least 2/3 of bacteria in bioaerosol constituted respirable fraction that migrates into the lower respiratory tract of the. The concentration of microbes inside animal rooms was higher as compared with the external environment, what means that rooms for animals were contaminated. Bacterial contamination inside animal facilities was particularly strong in spring and autumn, what is related to shedding fur by animals.

The analysis of isolated staphylococci revealed that *Staphylococcus succinus*, *S. sciuri*, and *S. vitulinus* are the most frequently occurring bacteria. Antibiotic susceptibility testing revealed that studied bacteria strains displayed the highest resistance to fusidic acid and rifampicin. The highest antibiotic resistance was noted in the case of *Staphylococcus epidermidis*, while the lowest for *S. xylosus.*

Taking into account the obtained results, necessary measures that may reduce the concentrations of undesirable microorganisms should be indicated. In addition to the daily cleaning of animal rooms involving, in particular, the removal of feces and replacement of litter, periodic disinfection of these rooms should be considered. Cleaning should be carried out as wet as possible, which should reduce dust and also the microbial concentrations in animal enclosures.

## Data Availability

The datasets used and/or analyzed during the current study are available from the corresponding author on reasonable request.
